# Filling Teeth

**Published:** 1872-02

**Authors:** E. G. Wheeler


### ARTICLE III.

### *Filling Teeth.*

BY E. G. WHEELER, D. D. S.

Read before the Alabama State Dental Association, August, 17th, 1871.

To the thoughtful and considerate practitioner, this subject can never become tame or uninteresting, because it is the important subject to which he has devoted his time and energies. It is the burden of his thoughts, and if successful, the crown of his rejoicing. We propose to take a cursory view of the subject, with the object before us, to call special attention to *errors* in practice, that failures may be avoided; and to advocate correct theories so far as we know, that success may be obtained

Let us enquire what constitutes *success* in filling teeth? We answer first, to preserve the tooth from further decay. Secondly, to restore partially or wholly its lost contour. It is not enough that fillings should not become loosened or drop out. The tooth should be put in such a thorough state of repair, that the chances for further decay is very much lessened, if not wholly destroyed. This cannot be done unless depressions and fissures leading out from the main cavity are thoroughly excavated and filled. It frequently takes more time and labor to explore a fissure leading from the central cavity in a molar tooth, than it would to fill a tooth in a careless and imperfect manner, and when four of these fissures occur, the operation becomes protracted, and sometimes complicated; yet *success* demands that each should be pursued to its terminus. A neglect of this precaution frequently renders it necessary to replace the filling or add others within a few months or years, when, if honestly and intelligently done at first, no after work would have been necessary.

But perhaps even the honest operator is called to mourn over failures, more frequently in approximal cavities in bicuspid teeth than in any other class. Perfect success in many of such cases, is in some degree uncertain, yet the operation can be much simplified, and the chances of success much increased by adopting the following plan, which we have found uniformly successful:

In case the decay occurs between the first and second inferior bicuspids, we use a common separating file, making a free opening to the bottom of the cavity, and continue filing until the approximal surface is clearly defined. Then use small enamel chisels, cutting down the crown immediately over the cavity, until you have an opening extending to the grinding surface, of about the same size as the cavity below. The excavation being thus made, the entire cavity becomes fully exposed to view. In shaping the cavity care must be taken to have it properly dove-tailed into the body of the tooth, so that the filling cannot be displaced. The filling of a tooth thus prepared becomes easy, as it requires only direct pressure in a line with the tooth. By this method, at least two important objects are gained. First, greater certainty of good results. Second, a saving of masticating surface, as it avoids those extrensive V shaped separations which are necessary in the usual process, also because the part cut away to gain access to the cavity, is replaced by the filling. The same process applies to approximal cavities in molar teeth, and with a greater prospect of success than by any other plan with which we are acquainted.

We may safely remark that many failures may be traced to a prejudice against, or timidity in cutting away sufficient of the tooth substance that may be necessary to render a complicated filling a simple one; and then again others have learned the lesson *too well*, who continue to cut and carve, until they mutilate what they should preserve. Both classes are *extremists*, and I doubt not that success lies between the two.

In the preparation of the cavity, we here lay down a few axioms or theorems, being the result of fifteen or twenty years practice, checkered ever as they are with various degrees of success, and failure. First, never attempt to fill a tooth until every part of the cavity can be seen, either with or without the aid of a glass. Second, always secure sufficient space, either by wedging, filing, chiseling, or extracting, so that you can have a free use of your instruments within the cavity. Third, avoid filling under a thin and exposed portion of enamel, lest it crumble off and destroy your filling. Better cut it away at once rather than incur the risk of losing your filling and perhaps your reputation. Fourth, attain a regular and firm margin to all cavities so that the union between the filling and tooth shall be complete, consequently no danger of leakage.

These are but simple rules, yet they cover much of the ground in which there are snares and pitfalls to entrap the unwary feet of the careless practitioner, and if honestly followed in the preparation of the cavity, much of the difficulty of filling is obviated, as we shall have by this time, secured self-confidence, as well as the confidence of the patient, both of which are essential to success.

But there are instances of excessively sensitive dentine, and of very nervous patients, when it becomes almost impossible to accomplish what we know to be essential to success. In such cases it becomes a matter of serious import what shall be done. We must resign the organ to destruction by dismissing the patient or perform an imperfect operation, unless we resort to remedial measures.

I cannot belive there is any rule that will apply to all cases, but as a general one, I believe it best to dismiss the patient rather than perform an imperfect operation. Still we may gain some advantages by temporary fillings, trusting to the benign effects of remedies, or of nature to remove the difficulties.

We might here also add to the causes of failure, imperfect and ill adapted instruments. Some dentists, either from penuriousness, ignorance, or old fogyism, are in this respect about as far behind this age of improvement as an ancient soldier would be with his matchlock or flint and steel gun; put some of the improved instruments into his hand, and he would be almost as much astonished at the ease and rapidity of the results attained, as an Egyptian would be at the discharge of a mitralieuse. As we pass from the preparation of the cavity to introducing the gold, the observation in reference to improved instruments is especially *apropos*, for, in this department, improvements have perhaps made the greatest strides, and one who is not up with the times and is ignorant of the advantages of such points as Atkinson's and Varney's, will very likely be found an incubus on the profession, crying down perhaps dental colleges, conventions, and all advancement generally.

In the preparation and introduction of the gold, there is more opportunity for display of one's originality and individuality, consequently the operator should be less trammelled by rule, excepting that of his own making, and it may not be very far from the truth when we say that many fail because they have no settled plan of their own, and never perfect any system which they from time to time may adopt. And yet one may change his theory and his practice several times during the course of twenty years, and still be entirely consistent, for while we should remember to hold fast to that which is good, we must not forget to press forward to "a higher mark," being careful, however, that all innovations on established rules shall be made cautiously.

There are many like myself, who once used nothing but soft gold, and now very little but adhesive gold, and yet have made the transit without violence or sudden change.

I first used adhesive gold to finish off with, and gradually increased it until now I seldom use anything else. In crown cavities I introduce the gold in the form of pellets, until the cavity is one-half or two-thirds full, consolidating at this point with mallet, then add layer upon layer of open leaf foil until the entire cavity is filled, using the heavier num bers to finish the filling. My success demands this course, while others may succeed as well or better, with soft, or semi-adhesive foil, prepared in the form of cylinders, or strips. But whatever is adopted, good caution is necessary to have the material properly consolidated, so as to resist the wear and tear of mastication. To do this it necessary to pack with small points, with direct pressure on every part of the filling. In filling approximal cavities when prepared as we have previously described, we commence by introducing cylinders as large as can be carrried to the bottom wall of the cavity safely, and continue this process until the cavity is half or two-thirds full, and then complete as in the case of crown fillings, care being taken that the cylinders shall be a little longer than the depth of the cavity, so that the filling will be flush on the approximal surface, which can be dressed down at pleasure. The use of adhesive foil enables the operator to overcome what I conceive to be a very common difficulty in the use of soft foil, viz: the inability to add to the surface of a filling when it was found to be not sufficiently full. But with adhesive foil it can be built up to any required height. The great enemy of adhesive foil, is *moisture*, and if not fully protected, failure is the result, but it is a failure which can be at once corrected, and does not immediately come to the dentist's ears, repeated by some kind friend after having passed the rounds of the social circle. But even while the work is being done judgement may be passed upon it with the utmost certainty. As a protection against moisture, nothing equals the rubber dam, it acts like a charm, it destroys the hydra-deaded monster (moisture) of all power to harm, and I hail it as one the most important inventions in modern dentistry, a worthy companion to the mallet and adhesive foil.

The discussions and and conflicting theories connected with the different modes of practice in operating upon teeth with exposed nerves, demonstrates the fact that we are still searching for truth. We need some *terra firma* upon which we may plant ourselves and say confidently "here we rest." We do not propose to review what has been said and written upon this subeject, but simply to give our mode of practice and the result, for what it may be worth, which from all the lights before us appears to be the best, because by this method we are the most successful and must submit to be called old fogy, and perhaps by harder names, by those who differ from us in practice. When I discover an exposed nerve, I consider first the age, constitution and temperament of my patient. If under twenty years of age and of a delicate constitution and nervous temperament, I almost despair of success except by destroying the nerve, still in very important teeth, a course of treatment to preserve the nerve alive, may be advisable, still in my practice such an effort is almost invariably attended with suppuration and loss of vitality sooner or later, and owing to this almost uniform result, I would feel unjustifiable in making the attempt, except as an experimental search after "more light,"

But with a less susceptible class of young patients, and most of those over twenty years, I regard the proper treatment of exposed nerves, with a view to preserving them alive, as not only justifiable but absolutely demanded of the dentist as the custodian of these important organs. We proceed first to apply creosote to remove inflammation and consequent pain, if the patient applies with an aching tooth, and after an interval of a day or two I excavate carefully as the soreness subsides, renewing the application of creosote or carbolic acid as may be necessary to restore the normal condition of the tooth, and to promote sloughing of the diseased portion of the nerve. Before filling, some advantage may be gained by placing a drop of collodion on the exposed nerve, and when evaporated proceed to fill the cavity with ox. chl. zinc in a soft paste, care being used to avoid pressure as far as possible. My own failures in this treatment, I attribute sometimes to hurrying the work too fast, not giving nature time to restore a healthy action, and perhaps this cause may operate with others to produce like results. Judgment must always be exercised in these cases as to the length of treatment and time for filling.

But when we chance to expose a healthy nerve in excavating, the treatment is easy and generally safe. Apply creosote for a few minutes, and fill immediately with ox. chl. zinc—when it becomes hardened all may be cut out excepting a portion over the nerve, and then filled with gold. But after all has been done, and science and art are exhausted, it is a very pertinent enquiry how many of these "perfectly successful operations" do not show dead nerves within ten years? If this perfected treatment, places the tooth in as complete a sanitary condition as before the nerve became exposed, why should this nerve be in any greater danger of dying than before it became exposed? That it is so, every practitioner can testify to his sorrow. Let us then not arrogate to ourselves, more than we deserve, but candidly admit that there are new discoveries to be made and higher attainments yet to be reached before we arrive at the perfection of nature.

That the chances of success are grerter in patients over twenty years of age, I regard as a fixed fact, but why this is so, can be more readily accounted for on the ground that certain pathological conditions are assumed about this age or soon after, which make suppuration less liable to occur. I arrive at this conclusion principally from the fact that the same course of treatment with patients under twenty almost always fails, attended with a great deal of annoyance, while my success has been most gratifying with those of more advanced age.

Taking everything together, it is questionable whether the profession has made much advancment beyond the well defined "land marks," of destroying nerves, and filling nerve cavity and crown. This is the *finale* of many a beautiful structure built over a pulsating nerve, when for a time all seemed to go "merry as a marriage bell." When I wish *certain results*, I destroy the nerve and subsequently fill.

I say *certain*, for by this course I have not had an abscess to occur, or necessity for the patient to return on account of serious trouble for several years. My practice is to apply the usual arsenious paste, allow it to remain from twenty-four to thirty-six hours; then remove, make a free opening into the pulp chamber, and apply cotton saturated with creosote, and dismiss the patient for a week unless necessity compels the work to be completed before— if so I see the patient every day, and continue treating with creosote or "phenol sodique," until the tooth is free from all soreness, and perfectly cleansed from all remains of the dead nerve. I then proceed to fill, commencing with a small piece of cotton moistened with creosote, which is forced to the very apex of the root, then commence with gold and complete the fang filling, and finish the crown filling the following day.

The object of delaying a week after the first application of creosote, is to allow time for the devitallized nerve to become partially dry and hardened, so that it can be removed whole. The points to be noticed in this process are first, remove every portion of the dead nerve. Second, remove soreness by application of creosote, "phenol," or tinct. camphor. Third, avoid doing too much at a time, allowing nature a chance to recuperate after the violent destruction of the vital part. Fourth, fill every portion perfectly. To destroy thermal influences, cover the fang filling with a layer of ox. chl. zinc, before putting in the crown filling. If every step is carefully taken, we cannot understand why it should ever give any further trouble, at least I am willing to risk my reputation on the result of such an operation, if carefully and intelligently done, rather than upon the most approved style of filling over exposed nerves, except in the most favorable cases.

In regard to finishing fillings, much time is frequently necessary to give proper shape and polish. But this becomes more a matter of taste than of real utility, if every step has been carefully taken thus far, although it must not be by any means neglected, if one would please those patients who are fastidious in matters of taste.

We do not propose to discuss in this paper, the effect of the surroundings in a dental office on success or failure, the mannerism of the operator, the necessity of cleanliness, or any other exterior influences which so much contribute to the final result, although it might be embraced within the scope of our subject. We prefer to leave these matters to be regulated by the gentlemanly instincts of the dentist, and if he has no such monitor to direct him, we shall gravely and silently conclude that he has mistaken his calling.

There remains the second portion of our definition of success to consider, viz : *Contour Filling.* To make this a success, the operator needs a quiet and sensible patient, the best adhesive foil in assorted numbers, rubber dam, an assistant to use the mallet and prepare gold as required, and a small alcohol lamp. When everything is ready, the cavity properly prepared. the rubber dam adjusted, the operation of filling commences, by first securing the base by means of grooves or pits into which the gold is packed securely, and when safely anchored, it is very easy to build up to any shape required.

By the aid of heavy foils, say No. 60 or 120, cut in small squares or strips, fillings can be built up very rapidly, and perhaps more securely than with lighter numbers. Every piece of gold should be placed over the flame of the alcohol lamp to remove any dampness which destroys its adhesiveness before it is packed. The malleting should be resorted to as often as necessary to condense perfectly, and when layer after layer is added and the filling assumes the proportions desired, the operator must feel a conscious pride at the beautiful structure which he has reared. But occasionally he may feel a tinge of trepidation lest this structure, though beautiful, may not be substantial; and I feel confident in saying that one who practices centour filling extensively, must expect some failures, for the class of teeth which require it are generally of a frail character, consequently operations upon them are liable to accidents. This being the case, to secure uniform good results, a just discriminatiion must be made between favorable and unfavorable subjects. The antagonism of the teeth must be carefully noticed. A projecting angle of the tooth may receive the whole force of the opposing tooth, and if not well supported may give way, and sometimes a sharp cusp may act as a wedge to loosen the filling. These peculiarities must be guarded against or failures will become so frequent as to discourage both patient and operator. The remedy in these cases may often be applied to the opposing tooth, in filing away a cusp or cutting an edge, so as to restore a perfect and natural antagonism.

We have now gone over some of the points which have suggested themselves to us connected with this subject, and have endeavored to contribute our mite of experience, and perhaps truth to the general fund. We cannot close without first remarking, that complete success rests much upon the fitness of the individual for his calling, first, by having the necessary talent, second, the proper education, and lastly, but not least, that moral courage which arises from a conscious honesty, and which enables him to look up and go forward with aspirations after higher attainments, and a determination to succeed. To all such I would say, be encouraged, go on seeking for the truth, and success will finally be your reward.

